# Inconsistent fertility motivations and contraceptive use behaviors among women in Honduras

**DOI:** 10.1186/1742-4755-6-19

**Published:** 2009-11-19

**Authors:** Ilene S Speizer, Laili Irani, Janine Barden-O'Fallon, Jessica Levy

**Affiliations:** 1 Department of Maternal and Child Health, Gillings School of Global Public Health, University of North Carolina at Chapel Hill, CB 7599, Chapel Hill, NC 27599-7599, USA; 2 MEASURE Evaluation Project, Carolina Population Center, University of North Carolina at Chapel Hill, 206 W Franklin Street, Chapel Hill, NC 27516, USA

## Abstract

**Background:**

Recent studies have demonstrated that it is common for women to report inconsistent fertility motivations and family planning behaviors. This study examines these inconsistencies among urban Honduran women interviewed at two points in time and presents reasons for inconsistent fertility motivations and contraceptive behaviors at follow-up.

**Methods:**

Data come from a one-year panel study conducted in Honduras from October 2006 to December 2007. A total of 633 women aged 15-44 years were interviewed at baseline and follow-up and have non-missing information on the key variables of interest. At baseline and follow-up, women were asked how much of a problem it would be (no problem/small problem/big problem) if they got pregnant in the next couple of weeks. At follow-up, women were asked an open-ended question on reasons it would be no problem, a small problem, or a big problem. The open-ended question was recoded into a smaller set of response categories. Univariate and bivariate analyses are presented to examine inconsistencies and reasons for stated inconsistencies.

**Results:**

At follow-up, over half the women using a contraceptive method said that it would be no problem if they got pregnant. Nearly half of the women changed their perceptions between baseline and follow-up. Common reasons for reporting no problem among contraceptive users were that they accepted a child as God's will or that children are a blessing, their last child was old enough and they wanted another child. Common reasons for reporting a big/small problem among non-users of family planning (who have an unmet need for family planning) were that they were not in a stable relationship, the husband was not present, and they would expect a negative response from their family.

**Conclusion:**

Inconsistent fertility motivations and contraceptive behaviors are common among effective contraceptive users. Women who are using contraception and become pregnant will not necessarily report the pregnancy as unintended, given the widespread acceptance of unintended pregnancies in Honduras. Family planning providers need to recognize that fertility motivations vary over time and that women may not have firm motivations to avoid a pregnancy.

## Background

Information on whether a woman wants to get pregnant soon, delay a pregnancy, or not have any (more) children is used to measure pregnancy intentions. Hence, an unintended pregnancy is defined as a pregnancy that is reported to have been unwanted (i.e., occur when no more children were desired) or mistimed (i.e., occur earlier than planned) [1]. Worldwide estimates of the proportion of unintended pregnancies are available through large scale population-based surveys such as the Demographic Health Surveys (DHS) and the Centers for Disease Control and Prevention Reproductive Health Surveys. In North America, nearly half (49%) of all pregnancies are reported to be unintended [2]. In Latin America and the Caribbean as well, around half of pregnancies are reported to be unintended [3]. In Honduras, for example, the most recent DHS data suggest that among births that occurred within the past five years, only half of them were desired by the mother at the time of the pregnancy [4]. Twenty six percent of Honduran women who gave birth in the past five years wanted to delay the pregnancy, while 24% had not wanted any more pregnancies. As a result, the wanted fertility rate was estimated to be 2.3 births per woman compared to the actual total fertility rate of 3.3 births per woman [4].

Many women who are not using contraception are at risk of an unintended pregnancy. A study from the U.S. demonstrated that among women who reported a pregnancy as unintended, about half were not using contraception at the time of pregnancy [5]. A number of studies from the U.S. have demonstrated that women may have ambivalent feelings about a pregnancy; that is, they may not feel strongly about whether or not to get pregnant soon (or ever). This ambivalence may affect use of a contraceptive method as well as the effectiveness of method use among those who are current users [6-9].

Studies from developing countries have also demonstrated that ambivalence towards pregnancy, and inconsistent fertility motivations and contraceptive use are common among contraceptive users and non-users. One study found that in Burkina Faso and Ghana, around 13% of women who wanted to delay or limit childbearing said that it would not be a problem if they became pregnant soon [10]. These findings were similar among contraceptive users and nonusers. In Kenya too, more than one-quarter of contraceptive users as well as nonusers who wanted to delay or limit childbearing gave an inconsistent response as to how much of a problem it would be if they got pregnant in the next few weeks [10]. Similarly, a study of female contraceptive users in Indonesia demonstrated that 5% of women said that it would not be a problem if they got pregnant in the next few weeks [Barden-O'Fallon J and Speizer I. Indonesian couple's ambivalence about a future pregnancy. Int Perspect Sex Reprod Health, in press]. On the other hand, among non-users of contraception, 52% of women said that it would be a small or a big problem if they got pregnant in the next few weeks; these women have an unmet need for family planning. Finally, a longitudinal study conducted in Morocco showed that among women who were not using contraception at baseline and who said that they did not want to get pregnant (and thus had an unmet need for contraception), two-thirds of those women who became pregnant between baseline and follow-up reported that they wanted the pregnancy [11]. Given these findings, worldwide rates of unintended pregnancies as well as the proportion of women who have a need for contraception based on their fertility motivations and non-use of contraception (unmet need) might be over- or under-estimated as the rates do not consider that many women may not have firm motivations toward a future pregnancy. Hence, the lack of firm motivations may affect use of contraception. It is also worth noting that several studies have shown that women who lack firm motivations towards a future pregnancy also may express ambivalence or be unsure about using contraceptives; these women may use less effective methods and be inconsistent users of methods [7-9,12-16]. To prevent unintended pregnancies, this ambivalence towards contraceptive use also needs to be acknowledged and addressed [17,18].

While quantitative data suggest that inconsistencies between fertility motivations and contraceptive use are common, few studies have attempted to determine the reasons for this ambivalence; the studies that have examined the reasons for these inconsistencies generally come from the U.S. [9,15,19,20]. However, using quantitative data, Bongaarts and Bruce examined DHS data from 13 countries and noted that the most common reasons for non-use of contraceptives among married women who did not wish to get pregnant were lack of knowledge of contraceptive methods, fear of side effects, disapproval from husband, and being opposed to family planning [12]. Less information is available from contraceptive users on their reasons for having inconsistent fertility motivations and contraceptive use behaviors. Moreover, besides the above mentioned study from Morocco that used longitudinal data, there is a lack of information from developing countries on the extent to which fertility motivations change over time and the circumstances of these changes.

This study fills these gaps by examining reasons given for fertility motivations among women who had inconsistent fertility motivations and contraceptive use behaviors using data from women in four cities of Honduras. Because the study collected two rounds of data (at baseline and one-year follow-up), it is also possible to examine the extent that fertility motivations change over the one-year follow-up period among women who were all using contraception at baseline and were predominately using their method to space their next birth.

## Methods

The data come from a panel study that examined contraceptive continuation among users of reversible female contraceptive methods in four major urban areas of Honduras: Tegucigalpa, San Pedro Sula, Santa Rosa de Copán/La Entrada, and Gracias. Data were collected at baseline and a follow-up interview took place one year later. Baseline data, collected between October-November 2006, were comprised of exit interviews with eligible women attending a family planning clinic to receive the oral contraceptive pill, injection, or intrauterine device (IUD). The selected clinics included seven Secretary of Health clinics (Centros de Salud Médicos Odontológicos or CESAMOs), one Secretary of Health hospital, and five Honduran Family Planning Association (Asociación Hondureña de Planificación de Familia or ASHONPLAFA) clinics. Eligible women were aged 15-44 years old, and were either new or continuing users of one of the above mentioned methods. All women who were eligible and visiting the target clinics during the study period were eligible for interview; no women refused to participate. At baseline, a total of 800 women participated in the study. Contact information, including addresses, maps, and directions to the women's homes, provided at baseline were used to locate the women and arrange for follow-up interviews. Follow-up interviews were conducted in October-December 2007. A total of 671 women (84%) from the baseline sample were found and interviewed at follow-up. Among those women who were not interviewed, 15% were not found and a small number refused (7 women) or had died (2). The comparison of those women who were successfully interviewed and those lost to follow-up revealed few differences between the groups [21].

At baseline, an interviewer asked women to respond to questions on their demographic characteristics, birth histories, previous use of contraception, perceptions of service quality, motivation to avoid pregnancies, and the family planning decision-making environment. At follow-up, women were asked about use of contraception during each month since the baseline interview, experience of and reactions to side effects, and updates on demographics, fertility motivations, and the decision-making environment. Ethical clearance was granted by the Institutional Review Board (IRB) of the University of North Carolina at Chapel Hill, the Honduran Secretary of Health, and ASHONPLAFA. Informed consent was obtained from each participant at the start of each interview.

At baseline and follow-up, women were asked to rate how much of a problem a pregnancy in the next few weeks would be for them. The close-ended responses were big problem, small problem, or no problem. At baseline and follow-up, women who were using effective contraception were considered to have inconsistent fertility motivations and contraceptive use behaviors if they responded that getting pregnant in the next few weeks would be no problem. Users of contraception gave consistent responses if they said that getting pregnant in the next few weeks would be a small/big problem. Women who were not using an effective contraceptive method at follow-up were considered to be inconsistent and have an unmet need for contraception if they responded that getting pregnant in the next couple of weeks would be a small/big problem. At follow-up, non-users of contraception were consistent and had no need for family planning if they said that getting pregnant in the next couple of weeks would be no problem. As a test, all analyses were run with the small-problem group included in the no-problem group. No changes were noted in the analyses, most probably due to the small sample size of the small-problem category. Responses at baseline were compared to follow-up.

As part of the follow-up questionnaire, women were also asked an open-ended question to provide, in their own words, a reason for why they reported that a pregnancy in the next few weeks would be no problem, a small problem, or a big problem. This question is used to provide a perspective on some of the factors that influence women's motivations to become or to avoid a pregnancy. Data on the open-ended question on why it would be no problem or a small/big problem if the woman becomes pregnant were recoded into a smaller number of categories. Categories were developed based on a cursory review of the data and additional categories were developed as needed. Two individuals (the first and second authors) recoded the data independently and their coding schemes were then compared (with the third author included) and when there were disagreements, the response was discussed and a consensus was achieved by the three-author team. Some women gave multiple reason responses; this happened among 112 of the women who responded to the open-ended question on their reason why getting pregnant would be no problem, a small problem, or a big problem.

The analysis sample was reduced from the full sample of 671 women included at follow-up because of missing data on the key variables of interest (the problem question and the open-ended question). In particular, 35 women were pregnant at the time of the follow-up interview and were not asked if becoming pregnant soon would be a problem. Of the remaining 636 women, three had missing data on reasons for why getting pregnant would be a problem. Therefore, 633 women gave reasons why getting pregnant soon would be no problem or a small/big problem; this is the analysis sample. Including the 112 women who gave two reasons, there are a total of 745 reasons included in the analyses. Descriptive analyses are presented including univariate and bivariate associations of the problem question and recoded open-ended question.

## Results

Table 1 describes some of the baseline socio-demographic characteristics of the 671 women interviewed at follow-up and the 633 women in the analysis sample. As shown in the table, there were no significant differences between the study population at follow-up and the analysis sample.

**Table 1:** Socio-demographic characteristics of the study population as assessed at baseline

	All women at follow-up		Analysis Sample*	
	N		N	
	671	(%)	633	(%)
Age				
19 or less	138	(20.6)	131	(20.7)
20-24	226	(33.7)	212	(33.5)
25-29	176	(26.2)	169	(26.7)
30-34	91	(13.6)	81	(12.8)
35+	40	(5.9)	40	(6.3)
Marital status				
In union	630	(93.9)	593	(93.7)
Not in union	41	(6.1)	40	(6.3)
Residential area				
Urban	537	(80.0)	509	(80.4)
Rural	134	(20.0)	124	(19.6)
Education				
None	39	(5.8)	36	(5.7)
Primary	433	(64.5)	404	(63.8)
Secondary+	199	(29.7)	193	(30.5)
Employed				
Yes	259	(38.6)	247	(39.0)
No	412	(61.4)	386	(61.0)
Parity				
0-1	296	(44.1)	280	(44.2)
2	187	(27.9)	173	(27.3)
3+	188	(28.0)	180	(28.5)
Contraceptive method				
Pill	46	(6.9)	41	(6.5)
Injectable	484	(72.1)	453	(71.6)
IUD	141	(21.0)	139	(21.9)
Problem if got pregnant now				
Big problem	350	(52.2)	332	(52.4)
Small problem	135	(20.1)	131	(20.7)
No problem	186	(27.7)	170	(26.9)

* Analysis sample includes all women with non-missing data on the open question.

About one-fifth of the women interviewed were less than 20 years old (Table 1) and 60% were between 20-29 years of age. The overwhelming majority (94%) of the women at baseline were in a union. Eighty percent of all respondents lived in an urban setting. Around 65% of all women had received some primary education while 30% had received some secondary education or beyond. A little less than half of the women had one or no child at baseline and about a quarter had two children and a similar percentage had three children. Notably, less than 1% of the sample had no children; this is why the no children and one child categories are combined. A little less than 7% were users of the contraceptive pill at baseline, almost 72% were using injectables, and the remaining 21% had an IUD. At baseline, 72% percent of women said getting pregnant in the next few weeks would be a small/big problem while 28% said it would not be a problem at all. The women were all using an effective method of contraception at baseline, thus the women who report that it would not be a problem are considered to have inconsistent fertility motivations at baseline.

Not surprisingly, many women changed their method use during the one-year follow-up period (not shown). Overall, at the end of the one-year period, 17% of women were no longer using a method (not shown). Women also switched between methods over the follow-up period with the most women switching away from injections and adopting another method (not shown). At follow-up, 47% of the analysis sample was using an injectable method, 18% had an IUD, and 11% were taking the pill. Table 2 shows that women's attitudes about pregnancy changed over the follow-up year. This table provides the comparison between baseline and follow-up responses as to how much of a problem a pregnancy in the next few weeks would be for the women in the analysis sample. Of the 332 women who at baseline said that getting pregnant soon would be a big problem, only 51% gave the same response at follow-up, while 40% switched to saying that it would be no problem if they became pregnant. A similar pattern is found among those who reported small problem at baseline. Hence, of the 463 women who said that it would be a small or big problem if they got pregnant at baseline, 212 (46%) switched their responses to no problem at follow-up. In contrast, of the 170 women who said that getting pregnant in the coming weeks would not be a problem at baseline, only 31% switched: 69% had the same response at follow-up, while the remaining women said a pregnancy in the next few weeks would be a big problem (22%) or small problem (9%).

**Table 2:** Responses to whether getting pregnant would be a problem, at baseline and follow-up

At baseline, how big a problem would it be to get pregnant	At follow-up, how big a problem would it be to get pregnant							
	
	Big problem		Small problem		No problem		Total	
	**N**	**%**	**N**	**%**	**N**	**%**	**N**	**%**
Big problem	169	(50.9)	30	(9.0)	133	(40.1)	**332**	**(100.0)**
Small problem	31	(23.7)	21	(16.0)	79	(60.3)	**131**	**(100.0)**
No problem	37	(21.8)	15	(8.8)	118	(69.4)	**170**	**(100.0)**

**Total**	**237**	**(37.5)**	**66**	**(10.4)**	**330**	**(52.1)**	**633**	**(100.0)**

In Table 3, we present the comparison between women's motivations to avoid a pregnancy and contraceptive use behaviors at follow-up. In this table, women who report that a pregnancy in the next few weeks would be no problem and that they are using a method of contraception at follow-up are considered to be inconsistent users of contraception. Fifty-three percent (n = 278) of the women using a contraceptive method reported that it would be no problem if they got pregnant and have inconsistent motivations and contraceptive use behaviors. Likewise, among women not using contraception, those women who report that a pregnancy in the next few weeks would be a big or small problem are considered to have inconsistent fertility attitudes and behaviors and have an unmet need for contraception. Fifty-two percent (n = 56) of the women not using a contraceptive method gave an inconsistent response and had an unmet need for contraception. Conversely, women who are using a contraceptive and report that the pregnancy would be a big or small problem are considered to be consistent with their family planning needs being met (met need).

**Table 3:** Response to whether getting pregnant would be a problem and contraceptive use, at follow-up

		Contraceptive method, at follow-up											
		
		Pill		Injectable		IUD		Other		Not using		Total	
		
		N	%	N	%	N	%	N	%	N	%	N	%
How big a problem would it be if got pregnant (follow-up)	Big problem	24	(34.3)	109	(36.6)	34	(30.1)	19	(43.2)	51	(47.2)	237	(37.5)
	Small problem	3	(4.3)	30	(10.1)	23	(20.3)	5	(11.3)	5	(4.6)	66	(10.4)
	No problem	43	(61.4)	159	(53.3)	56	(49.6)	20	(45.5)	52	(48.2)	330	(52.1)
	**Total**	**70**	**(100.0)**	**298**	**(100.0)**	**113**	**(100.0)**	**44**	**(100.0)**	**108**	**(100.0)**	**633**	**(100.0)**

In Tables 4 and 5, the analysis focuses on the reasons given for reporting no problem, small problem, and big problem based on the above identified categories of consistent/inconsistent and use/non-use. The number of women is smaller than the number of responses since some women gave two reasons. Among the 330 women who reported at follow-up that getting pregnant would be no problem, there were 353 reasons given to why it would not be a problem (23 women gave two answers). The reasons why getting pregnant would be no problem for all women are presented in the first column of results in Table 4. The most common reason for reporting "no problem" at follow up was coded as "Acceptance" which included answers such as "What happens, happens, I can't do anything but accept it. What can I do?" and "We accept what comes, whatever it is, because you don't deny children." The next most common type of response was "God's will," suggesting that if the woman gets pregnant even while using contraception, it would not be a problem because it was what God wanted. The only other answer that attained more than 10% of the responses was that it would not be a problem because the last child was old enough.

**Table 4:** Reasons why getting pregnant would be no problem, at follow-up

Reasons cited	Total responders		Consistent responders/Non-users		Inconsistent responders/Users	
	
	All women who responded 'no problem' at follow-up		Women who responded 'no problem' and were not using a method		Women who responded 'no problem' and were using a method	
	N	(%)	N	(%)	N	(%)
Acceptance	97	(27.5)	10	(18.5)	87	(29.1)
No other option						
That is what it means to be human						
What God wants	86	(24.4)	5	(9.3)	81	(27.1)
Children are a blessing	27	(7.6)	4	(7.4)	23	(7.7)
Must love all children equally						
It would be wonderful						
Children are always welcome						
Last child is old enough	39	(11.0)	7	(13.0)	32	(10.7)
Resources are available	12	(3.4)	1	(1.8)	11	(3.7)
Can afford it						
Has a house						
Planning a family	25	(7.1)	9	(16.7)	16	(5.4)
Wants/desires another child						
Better to have children young/quickly						
Partner wants to have a child	10	(2.8)	3	(5.5)	7	(2.3)
We both want to have a child	17	(4.8)	7	(13.0)	10	(3.3)
Partner is present	12	(3.4)	2	(3.7)	10	(3.3)
Would make husband happy	2	(0.6)	0	(0.0)	2	(0.7)
Other	20	(5.7)	6	(11.1)	14	(4.7)
Not applicable	6	(1.7)	0	(0.0)	6	(2.0)

**Total**	**353**	**(100.0)**	**54**	**(100.0)**	**299**	**(100.0)**

Note: the number of women (presented in the text) is smaller than the number of responses because some women gave multiple responses.

**Table 5:** Reasons why getting pregnant would be a big/small problem, at follow-up

Reasons cited	Total responders		Consistent responders/Met need		Inconsistent responders/Unmet need	
	
	All women who responded 'big/small problem' at follow-up		Women who said big/small problem and were using a contraceptive method		Women who said big/small problem and were not using a contraceptive method	
	N	(%)	N	(%)	N	(%)
Last child is still young	110	(28.1)	96	(29.6)	14	(20.6)
Economic situation is not good or high cost of living	59	(15.1)	52	(16.1)	7	(10.3)
Respondent is studying/working	44	(11.2)	42	(13.0)	2	(2.9)
Desire to give other children attention, support, love	12	(3.1)	11	(3.4)	1	(1.5)
Medical complications	25	(6.3)	22	(6.8)	3	(4.4)
Doctor advises against it						
Respondent has general medical problems						
Past pregnancy complications						
Finished childbearing	32	(8.1)	31	(9.6)	1	(1.5)
Already too many children						
Other children are too old- does not want to start over Old age						
Having a family is not in plans	22	(5.6)	17	(5.2)	5	(7.3)
Respondent does not want to get pregnant						
It would be something unexpected/not in plans						
Not in stable relationship, or no partner	18	(4.6)	11	(3.4)	7	(10.3)
Husband is not living with woman	12	(3.1)	1	(0.3)	11	(16.2)
No one is available to help raise the child (e.g., a single parent, no family support for childcare)	14	(3.5)	9	(2.8)	5	(7.4)
Negative response from extended family	12	(3.1)	5	(1.5)	7	(10.3)
Problem with woman's own or partner's family (e.g., may get upset)						
Living with parents/in-laws						
Other	16	(4.1)	14	(4.3)	2	(2.9)
Not applicable	16	(4.1)	13	(4.0)	3	(4.4)

**Total**	**392**	**(100.0)**	**324**	**(100.0)**	**68**	**(100.0)**

Note: the number of women (presented in the text) is smaller than the number of responses because some women gave multiple responses. Unmet need means that the woman is sexually active and reports that she does not wants to get pregnant but is not currently using an effective method of contraception; met need means that the woman does not want to get pregnant and is using an effective contraceptive method.

Table 4 also presents the results by whether the woman is consistent in her report of fertility motivations and contraceptive use. There were 52 women (or 16%) who were not using a contraceptive method and reported a pregnancy would be no problem; this is considered a consistent response. Women who were consistent were more likely to report that they wanted to get pregnant or they were planning a family. They also gave the acceptance type responses. This group was much less likely to report religious responses. The other group of women presented in Table 4 is the women who gave inconsistent responses, that is, those women who reported that a pregnancy would be no problem but were using a method of contraception at follow-up; this was the larger group. Among the 278 women in the inconsistent/user category, 8% gave multiple answers. These women were more likely to report acceptance and God's will type answers as well as that the last child was old enough.

Of the 303 women who said that getting pregnant soon would be a big/small problem, 82% were using a contraceptive method at the time of the interview; this is a consistent response (consistent/met need). On the other hand, 19% of the women were not using a contraceptive method, even though they stated that getting pregnant soon would be a big/small problem. These women have an unmet need for contraception, and they are inconsistent in their fertility desires and contraceptive behaviors. The most common reasons cited for why a pregnancy soon would be a small/big problem among the women with an unmet need for contraception (Table 5) were that their last child was still young (20%), and that their husband/partner was currently not living with them (16%). Other reasons stated were that they were not in a stable relationship (10%), they were afraid of a negative response from their parents/in-laws if they got pregnant (10%), their economic situation was not stable enough for them to raise a child (10%), or that their partner/extended family was not available to help them raise another child (7%). Additional reasons and the frequency of responses are presented in Table 5.

Among women with consistent contraceptive behavior and fertility motivations, the main reasons cited for why getting pregnant would be a big/small problem were that the last child was still young (30%), that the family's economic situation was not adequate to have another child (16%), or that the woman was at that time studying/working (13%). Other reasons cited for why getting pregnant would be a big/small problem were that the woman had finished childbearing (10%) or that she was unable to have another child due to past/current medical complications (7%).

## Discussion

This study demonstrates that 27% of the sample of urban Honduran women who were using the IUD, injection, or the pill at baseline reported that it would be no problem if they became pregnant in the next few weeks. These women have inconsistent fertility motivations and contraceptive use behaviors at baseline. At one-year follow-up, 17% of the sample was no longer using an effective method of contraception and only 59% were using their baseline method. The extent of changes in contraceptive method use between baseline and one-year follow-up is indicative of potential problems with the methods (e.g., side effects, problems with access) as well as changing pregnancy desires in the period. Notably, about half of the women who were not using a contraceptive method at follow-up have an unmet need for family planning; that is, they reported that it would be a big/small problem if they got pregnant in the next few weeks. At follow-up, we also found that more than four-fifths of women who report that getting pregnant in the next few weeks would be no problem were effective method users; these women are considered to have inconsistent fertility motivations and behaviors. The most common reasons for reporting no problem among these women were that they would accept the pregnancy, children are God's will, and that children are a blessing and are always welcome. Conversely, the women who reported that a pregnancy in the next few weeks would be no problem and were not using contraception (a consistent response) tended to give reasons related to motivations to get pregnant.

This study further demonstrates that 19% of the women interviewed who are not using any contraceptive method report that it would be a big/small problem if they became pregnant in the next few weeks. These women are considered to have an unmet need for contraception. Among these women, the most common reasons for why a pregnancy would be a big/small problem are related to partner and family issues. On the other hand, more than four-fifths of the women who were using a contraceptive method at the time of the follow-up interview gave a consistent response that it would be a big/small problem if they became pregnant in the next few weeks. These women gave reasons related to their economic and educational situations as well as their plans to delay a pregnancy. These results illustrate that women who are consistent in their contraceptive behaviors and fertility desires appear to plan their pregnancies, whereas the women with an unmet need for contraception demonstrate partner and family support concerns that influence their motivations for a future pregnancy.

Most studies on fertility desires focus on retrospectively reported pregnancy intentions or examine which women have an unmet need for contraception based on their stated fertility desires [8,22,23]. This study demonstrates that even users of three effective contraceptives may provide inconsistent answers to fertility motivations and contraceptive use behaviors. Our study also builds upon previous work on fertility intentions by including a novel question on how much of a problem it would be to become pregnant in the next few weeks [24]. Schwarz and colleagues [7] demonstrated that when women are offered a small number of response options for fertility intentions, they do not appear ambivalent; however, when response options are expanded, there is greater ambivalence about future childbearing. Our findings confirm the Schwarz findings. By including a novel measure of how much of a problem it would be to become pregnant and comparing it to contraceptive use, we find that about a quarter of women provide inconsistent responses at baseline. Moreover, by including the reasons women give for why a pregnancy in the next few weeks would be no problem, a small problem, or a big problem, this study permits a greater understanding of motivations to avoid a pregnancy among users and non-users of contraception. Finally, an additional strength of this study is that fertility desires are measured over time. This helps demonstrate that pregnancy intentions vary with time, even within a time period as short as one year.

A limitation of this study is that we cannot compare over time the reasons women gave as to why getting pregnant would be a big, small, or no problem as this information was only collected at follow-up. Also, while we report on inconsistencies between fertility motivations and contraceptive use behaviors, we may in part be measuring ambivalence toward contraceptive use rather than problems with the meaning or measurement of fertility motivations [9]. Contraceptive ambivalence may reflect experience with side effects, health concerns, distrust of methods, or religious beliefs against contraception. An additional limitation is that women were asked to give a reason for why a pregnancy would be no problem, a small problem, or a big problem. Most women gave one reason while some gave two. Although the reasons given provide an understanding of women's thought processes around fertility decision-making, it is important to note that the women are only giving the first reason that comes to their heads and this may not be the most important reason. More in-depth qualitative data collection is needed to obtain a broader list of reasons and the level of importance assigned to each reason. This was beyond the scope of this study. Finally, it is possible that women who were using a hormonal contraceptive method reported no problem because they are using this method for problems with menstruation and not as a family planning method. With the data available, it is not possible to tease out which women are in this category.

Our study demonstrates important findings for family planning programs. First, just examining standard fertility intentions - wants now, wants to delay, wants no more - will not provide an accurate prediction of who needs or will use long-term effective methods. Second, we note that fertility intentions can change over time. Third, women who are using contraception and have inconsistent fertility motivations with their current use behaviors are more likely to accept a future pregnancy as the will of God and raise the child as a blessing they need to nurture. Hence, the outcome of an unintended pregnancy may be an intended birth. Programs may need to focus on giving women more autonomy and confidence when planning a family to ensure they are using a method that meets their current fertility intentions. Furthermore, women who have an unmet need for family planning and report that a pregnancy in the next few weeks would be a big problem may need to be counseled about the advantages of consistent and effective method use to avoid an unintended pregnancy.

## Conclusion

This study demonstrates that many effective family planning users have inconsistent fertility motivations. Future studies are needed to examine whether less motivated women are more likely to discontinue use when they experience partner or family opposition; side effects; or changes to their economic or educational situation. Qualitative studies are also needed to determine whether the most motivated women are receiving the methods that are best suited to their fertility desires. Among women who use temporary methods of contraception, even though they might state no intention of a future pregnancy (or a desire to delay a pregnancy), they may not have strong desires to avoid pregnancy and this might relate to their method choice or the effectiveness of method use. Family planning program officers need to be aware that women's fertility intentions can change within a short period of time, even though they might continue to use a contraceptive method. Moreover, if a pregnancy happens, it will not necessarily be reported as unintended, given that most births would be accepted among women in Honduras. Family planning program managers should consider strategies to ensure that motivated users have access to follow-up care, if needed, to address method concerns such as side effects. Finally, a greater understanding of fertility motivations and how they influence the effectiveness of contraceptive use is needed to help family planning providers ensure that they are counseling women appropriately and could help reduce the prevalence of unintended pregnancies in Honduras and other countries where unintended pregnancies are common.

## Competing interests

The authors declare that they have no competing interests.

## Authors' contributions

ISS conceived of the idea, participated in data coding, analysis, and writing. LI led data coding and analysis and revised the manuscript. JB participated in data coding, analysis, and writing. JL participated in data collection and contributed to paper development. All authors read and approved the final manuscript.

## References

[B1] Santelli J Rochat R Hatfield-Timajchy K Gilbert BC Curtis K Cabral R Hirsch JS Schieve L Unintended Pregnancy Working Group The measurement and meaning of unintended pregnancy Perspect Sex Reprod Health 2003 35 94 101 10.1363/3509403 12729139

[B2] Finer LB Henshaw SK Disparities in rates of unintended pregnancy in the United States, 1994 and 2001 Perspect Sex Reprod Health 2006 38 90 96 10.1363/3809006 16772190

[B3] Macro International Inc MEASURE DHS STAT Compiler 2009 http://www.measuredhs.com

[B4] Secretaría de Salud [Honduras], Instituto Nacional de Estadística (INE), International M Encuesta Nacional de Salud y Demografía 2005-2006 Tegucigalpa, Honduras: SS, INE y Macro International 2006 440

[B5] Brown SS Eisenberg L Committee on Unintended Pregnancy: Chapter 4. Patterns of Contraceptive Use The Best Intentions: Unintended Pregnancy and the Well-Being of Children and Families 1995 Washington D.C.: National Academy Press Press NA (Series Editor) 25121228

[B6] Luker KC A reminder that human behavior frequently refuses to conform to models created by researchers Fam Plann Perspect 1999 31 248 249 10.2307/2991574 10723651

[B7] Schwarz EB Lohr PA Gold MA Gerbert B Prevalence and correlates of ambivalence towards pregnancy among nonpregnant women Contraception 2007 75 305 310 10.1016/j.contraception.2006.12.002 17362711

[B8] Trussell J Vaughan B Stanford J Are all contraceptive failures unintended pregnancies? Evidence from the 1995 National Survey of Family Growth Fam Plann Perspect 1999 31 246 247 260 10.2307/2991573 10723650

[B9] Zabin LS Ambivalent feelings about parenthood may lead to inconsistent contraceptive use--and pregnancy Fam Plann Perspect 1999 31 250 251 10.2307/2991576 10723653

[B10] Speizer IS Using strength of fertility motivations to identify family planning program strategies Int Fam Plan Perspect 2006 32 185 191 10.1363/3218506 17237015

[B11] Bankole A Westoff CF The consistency and validity of reproductive attitudes: evidence from Morocco J Biosoc Sci 1998 30 439 455 10.1017/S0021932098004398 9818553

[B12] Bongaarts J Bruce J The causes of unmet need for contraception and the social content of services Stud Fam Plann 1995 26 57 75 10.2307/2137932 7618196

[B13] Crosby RA Diclemente RJ Wingood GM Davies SL Harrington K Adolescents' ambivalence about becoming pregnant predicts infrequent contraceptive use: a prospective analysis of nonpregnant African American females Am J Obstet Gynecol 2002 186 251 252 10.1067/mob.2002.120081 11854644

[B14] Frost JJ Singh S Finer LB Factors associated with contraceptive use and nonuse, United States, 2004 Perspect Sex Reprod Health 2007 39 90 99 10.1363/3909007 17565622

[B15] Iuliano AD Speizer IS Santelli J Kendall C Reasons for contraceptive nonuse at first sex and unintended pregnancy Am J Health Behav 2006 30 92 102 16430324 10.5555/ajhb.2006.30.1.92

[B16] Schunmann C Glasier A Measuring pregnancy intention and its relationship with contraceptive use among women undergoing therapeutic abortion Contraception 2006 73 520 524 10.1016/j.contraception.2005.12.009 16627038

[B17] Curtis SL Blanc AK Determinants of contraceptive failure, switching, and discontinuation: An analysis of DHS contraceptive histories, DHS Analytical Reports No. 6, Calverton, MD: Macro International Inc 1997

[B18] Kost K Singh S Vaughan B Trussell J Bankole A Estimates of contraceptive failure from the 2002 National Survey of Family Growth Contraception 2008 77 10 21 10.1016/j.contraception.2007.09.013 18082661 PMC2811396

[B19] Stevens-Simon C Kelly L Singer D Cox A Why pregnant adolescents say they did not use contraceptives prior to conception J Adolesc Health 1996 19 48 53 discussion 54-45 10.1016/1054-139X(95)00281-V 8842860

[B20] Stevens-Simon C Sheeder J Harter S Teen contraceptive decisions: childbearing intentions are the tip of the iceberg Women Health 2005 42 55 73 10.1300/J013v42n01_04 16418122

[B21] Barden-O'Fallon J Speizer I Zelaya SC Borjas JC Valenzuela FR Contraceptive discontinuation: a one-year follow-up study of female reversible method users in urban Honduras 2008 Chapel Hill, NC: MEASURE Evaluation 45

[B22] Speizer IS Santelli JS Afable-Munsuz A Kendall C Measuring factors underlying intendedness of women's first and later pregnancies Perspect Sex Reprod Health 2004 36 198 205 10.1363/3619804 15519962

[B23] Westoff CF Unmet Need at the End of the Century DHS Comparative Reports No 1 2001 Calverton, MD: ORC Macro

[B24] Schaeffer NC Thomson E The Discovery of Grounded Uncertainty: Developing Standardized Questions about Strength of Fertility Motivation Sociological Methodology 1992 22 37 82 10.2307/270992

